# A Medical Causerie

**Published:** 1915-06-26

**Authors:** 


### A MEDICAL CAUSERIE.

The Jonathan Hutchinson Clinical Museum, which was formerly housed at the London Polyclinic, and which, as we intimated some weeks ago, is to be transferred to the Johns Hopkins Hospital, Baltimore, was secured for this latter institution by the generosity of Mr. W. A. Marburg, who has previously shown his interest in the great post-graduation school of the United States. In a preliminary note on the collection Sir William Osler emphasises its wide range and great teaching and clinical value. While the illustrations relating to skin diseases and to syphilis are the most numerous, there is scarcely a department of either medicine or surgery which fails to be represented in the museum. In speaking of the manner of utilising these pictorial illustrations of diseased conditions Sir William Osler insists upon the necessity of making them freely accessible to the medical student, and points out their great value as aids to clinical demonstration. He endorses in so many words the views expressed in The Hospital of May 8. Some years ago Sir Jonathan offered his collection to the Royal College of Surgeons on the condition that it should be displayed and not hidden away in cabinets and drawers. The offer was declined, and no British medical school has had sufficient enterprise to purchase the museum when it came into the market. Now it has obtained a Transatlantic home and is sure of the full appreciation which it never received in the country of its origin. To take a single department only, the museum is described on unquestioned authority to be " probably the most remarkable iconography on syphilis ever made."

The recent elections for the Council of the British Medical Association appear to have attracted a mere minimum of interest. In the several branches throughout the kingdom there were no contests except in the London area. Here there were five candidates for four vacant seats, and the total number of votes polled was 591, out of a constituency which includes several thousand members. At the head of the poll were two consulting physicians, and these were followed by two general practitioners. The election was conducted on the principle of proportional representation, but the result would apparently have been the same had the ordinary method of voting been followed. Not much has been heard of the London War Emergency Committee's operations. Nevertheless, it has been doing substantial work. The information obtained by its census of the Metropolitan area is likely to be of great service to the War Office, as it displays not only the number of doctors available for service, but also the difficulties hindering many from giving the help which on patriotic grounds they would desire to offer. These difficulties once known, it will rest with the authorities to remove them if they really need assistance.

A story recently to the fore illustrates how the invasion of the R.A.M.C. by numbers of civil practitioners may at times put a strain upon the official bonds of the Army organisation. A well-known physician on service in one of the military hospitals, and having under his care a case of mitral disease, marked the diagnosis sheet with the term " auricular fibrillation." Promptly it was returned with a demand for revision on the ground that no such term as " auricular fibrillation " had a place in the official terminology relating to diseases of the ear! The new wine of the polygraph is evidently dangerous to the old bottle of the military clinician. Diagnosis must be " by the card, or equivocation will undo us."

At the recent " Presentation Day " of the University of London the Chancellor, Lord Rosebery, in referring to the influence of the war, said that its effect on University teaching was disastrous. Not only were the numbers of the students diminished and the income depleted, but in view of the tremendous drain upon the national exchequer it was now impossible to hope that the Government would be able to provide any such generous endowment and palatial home as had been anticipated. In view of this position harmonious working of the different interests is all the more important, and the Chancellor appealed for a recognition of the necessity of development alike for the external as for the internal side of the University. Both the one and the other are essential if a full measure of success is to be realised. The new Vice-Chancellor is Sir Alfred Pearce Gould, K.C.V.O., who succeeds Sir Wilmot Herringham, now serving with the Army in France. It is only too certain that the report of the latest of the numerous Royal Commissions which have " sat upon " the University of London will remain for long upon the shelf. Whatever be its merits, circumstances are against it. Thus the present system of medical teaching in the Metropolis, though often threatened in certain important respects, is likely to survive the latest assault, and this quite apart from its merits or defects. Before " reform " can again appear in the air, circumstances and the point of view will have changed, and the Haldane Report will merely be a historical document sleeping with a goodly row of predecessors in the dust of some subterranean Government department.

A somewhat energetic patriotism has recently been displayed by one of the suburban hospitals. Having in its service as resident medical officer a young lady who, while herself a British subject, was descended from an alien enemy, the authorities decided summarily to dismiss her. No personal or professional delinquency was alleged, and presumably the step was taken as a sop to the " public opinion " of the local subscribers.

The British Fire Prevention Committee has issued a useful warning against the powder extinguishers, hand grenades, and similar contrivances which are largely boomed as certain methods of extinguishing fires likely to be caused by incendiary bombs. The prompt application of water in fair bulk, force, and continuity is the right procedure to adopt. A series of useful directions and hints is published by the Committee, and can be obtained on application at 8 Waterloo Place, S.W.

